# Smoke-free-school-hours at vocational education and training schools in Denmark: attitudes among managers and teaching staff – a national cross-sectional study

**DOI:** 10.1186/s12889-019-7188-0

**Published:** 2019-06-24

**Authors:** Clara Heinze, Anneke Vang Hjort, Peter Elsborg, Helle Terkildsen Maindal, Charlotte Demant Klinker

**Affiliations:** 1 Health Promotion, Steno Diabetes Center Copenhagen, Niels Steensens vej 6, 2820 Gentofte, Denmark; 2 Department of Public Health, Aarhus University, Bartholins Alle 2, 8000 Aarhus, Denmark

**Keywords:** Attitudes, Smoking cessation, Vocational schools, Cross-sectional study, Denmark, Health promotion

## Abstract

**Background:**

Tobacco is the main cause of non-communicable disease and premature death globally. Implementing restrictive school tobacco policies such as smoke-free-school-hours (SFSH) may have the potential to reduce smoking among Vocational Education and Training (VET) school students. To be effective, school tobacco policies that largely involve strict and consistent enforcement by both managers and teaching teaching staff must be implemented. This study investigated the attitudes towards the implementation of SFSH among the managers and teaching staff at Danish VET schools.

**Methods:**

The analyses were based on cross-sectional survey data collected with an online survey among managers and teaching staff at Danish VET schools. The data was collected from March to June 2017.

**Results:**

Managers and teaching staff (*n* = 571) from 71 out of 87 Danish VET schools (81.6%) took part in the survey. In the adjusted analysis, teaching staff were twice as likely as managers to have a favourable attitude towards SFSH. Furthermore, being female and of increasing age correlated with having a favourable attitude. A trend towards schools in favour of SFSH having more health promotion facilities, policy and practice, was identified.

**Conclusion and implications:**

Existing health promotion facilities and activities at the schools were associated with a favorable attitude among the management towards implementing SFSH. Thus, implementing other health promotion activities and policies might be an important first step to establish readiness to implement SFSH.

## Background

Tobacco is the main cause of non-communicable diseases and premature death globally [1–3], and tobacco use is a global burden with massive costs in terms of both quality of life and individual- or national healthcare expenses [2].

In European countries, Vocational Education and Training (VET) school students are more likely to be daily smokers compared to students with a higher education [4, 5]. This is also the case in Denmark, where the prevalence of daily smoking among students enrolled in VET schools is 37% compared to 12% among peers in high schools [6]. Students enrolled in VET to a large extent come from low socioeconomic status groups [7, 8]. VET schools have been found to be an important setting for health promotion to target inequality in health [9, 10] and more specifically to target smoking prevention and cessation [4, 9].

As part of the World Health Organization (WHO) treaty Framework Convention on Tobacco Control (FCTC), it is recommended that children, youth and adults be protected from exposure to tobacco by implementing smoke-free policies [2]. A number of studies have found that strict and consistent enforcement of tobacco policies, at both school and work settings, may prevent or reduce cigarette smoking [11–14].

Due to the ‘Smoke-Free Environment Act’ of 2012, smoking within Danish schools and educational institutions where most students are under the age of 18 years is prohibited [15, 16]. As the majority of students are over 18 years old in VET schools, the current legislation does not apply for VET schools [16, 17]. Instead, VET schools are obliged to formulate autonomous policies for tobacco use within school hours and on school premises [18], and thus it is the management’s decision as to whether to implement smoke-free-school-hours (SFSH) [17].

The concept of SFSH bans the use of any tobacco-related products (e.g., e-cigarettes, “heat-not-burn” products or snuff) during school hours – both inside and outside the school premises – and it covers managers, teaching staff, students and guests. SFSH is thus a more comprehensive tobacco policy than the current legislation. SFSH is a relatively new concept, and evidence of its effects is sparse [19, 20]. For example, Norway’s smoking bans, and restrictions are almost in compliance with the WHO FCTC recommendations and have established SFSH in all educational institutions, including VET schools. Promising data show a smoking prevalence for Norwegian youth (individuals aged 16–24) of 3% in 2017, compared to 16% among Danish youth in 2017 [20].

The decision to adopt smoke-free policies in VET schools is more likely to be successfully implemented if there is support from both management and teaching staff [21–23]. As such, attitudes towards tobacco policies among managers and teaching staff at VET schools may serve as a proxy for the likelihood of the schools’ readiness to implement more restrictive school policies such as SFSH.

In 2015, the latest Danish VET school reform was ratified, introducing health promotion as part of school practice: 45 min daily exercise for all students, health education for the youngest students and an overall focus on increasing student well-being [24]. The rationale was to increase vocational performance and reduce student drop-out as evidence shows that health and wellbeing are correlated with drop-out and later work capacity in the labour market [25]. As such, a responsibility for students’ health and well-being was placed with the schools [26]. When schools begin to acknowledge and embrace the role they can and shall play in health promotion in relation to their students physical activity and wellbeing - as enforced by the VET school reform - this might influence their attitude towards health promotion in other areas, such as smoking, and it may encourage more pioneering schools to work more generally with health promotion initiatives and perhaps implement SFSH.

Little is known about managers’ and teaching staffs’ attitudes towards implementing SFSH, and the objectives of this study were first to assess attitudes towards implementing SFSH among managers and teaching staff at Danish VET schools, second to examine what is associated with managers’ and teaching staffs’ attitudes towards implementing SFSH, and third to explore the differences in health promotion environments among VET schools based on management’s attitudes towards SFSH.

Based on recent studies [23, 27–29], we expected that VET school management would have a more favourable attitude towards implementing SFSH compared to the teaching staff. Moreover, we hypothesized that the management had a more favourable attitude towards implementing SFSH on schools with a higher focus on health-promoting initiatives, compared with schools where health-promotion were less in focus.

## Methods

### Study design and setting

This paper is based on data from a cross-sectional survey that aimed to map health promotion policies and practice in all Danish VET schools as well as investigate managers’ and teaching staffs’ attitudes in relation to same [29]. After 9 years of primary and lower secondary school, the majority of young people in Denmark choose to continue into the academically oriented upper secondary schools system (56%) or VET schools (23%), which include, e.g., agricultural, commercial, technical, or social healthcare programmes [30]. In Denmark, there is a large variation in the age of students in VET schools, as some students continue directly from primary school to VET school (age 15–17 years old) while others enrol a VET programme later in adult life (44.5% > 25 years old) [31]. The average age of a student enrolled in a VET programme is 24 years [32]. The VET programmes vary in length but last an average of 3 to 4 years. The programme combines alternating school-based training (one-third) and workplace-based training (two-thirds) [8, 33].

In 2017, Denmark had 87 VET schools with more than 100.000 students enrolled (18.5% of all youth in Denmark) [31]. The schools are located in all five Danish regions but mainly in the larger cities [34]. Some Danish VET schools have several departments located at different physical addresses and across different municipalities. The mean student population is 1150 students per school with a range of 200 to 7500 students [35]. Most VET schools are organized with one or two principal managers, some department managers and teaching staff. The principal manager has the overall management and operational responsibility for the entire institution, and the department managers have management responsibility for either a specific department or an educational programme. The teaching staff have daily contact with the students and are directly responsible for delivering the curriculum [33].

### Data collection

The data were collected from March to June 2017 using an electronic questionnaire distributed to principal managers, department managers and teaching staff. Since the survey was designed for another purpose no sample size calculation was made. Initial contact with all 87 Danish VET schools was established by email and by telephone with the principal manager. If the principal manager accepted that the managers and teaching staff could take time off to participate in the study, the applied recruitment strategy was to recruit one principal manager and ask him or her to select a representative sample of department managers who would then recruit a representative sample of at least five teaching staff members based on predefined criteria. The criteria were 1) different attitudes and experiences in relation to health promotion activities, 2) teaching different educational programmes and 3) difference in time length of employment. If this was not possible due to busy school schedules, only the principal manager was asked to participate in the survey.

All recruited participants received an e-mail with a personal link to the electronic questionnaire system (SurveyXact). The e-mail also included a description of the study’s aims and methods and a statement to the effect that participation was anonymous and voluntary and that the participants could withdraw at any time without any repercussions. Four reminder rounds were conducted (three electronic requests and one by telephone) to the recruited participants who had not yet answered the questionnaire.

The questionnaire did not encompass personally sensitive data; thus, no institutional ethical approval was obtained as this is not a requirement for social science research in Denmark [36]. Participation was voluntary, and a disclosure of confidentiality was provided, along with a statement of adherence to The Danish Data Protection Agency rules and regulations.

### The questionnaire

The questionnaire consisted of both items that were the same across the three respondent groups (principal managers, department managers and teaching staff) and items that were tailored to the specific respondent group. Approximately 33% of the items were the same for all three respondent groups. The principal management level was asked about the institution’s strategic work, health promotion policies and activities across the organization. The department managers were asked about the specific department’s health promotion policies and activities. The teaching staff were asked about actual health promotion practices and the integration of health promotion into the school curriculum. The questionnaire was piloted within the target group (management and teaching staff in VET schools) and in a reference group consisting of people with knowledge and expertise on the Danish VET school system and/or health promotion surveys in general. The content of the questionnaire was based on inputs from the reference group as well as the Danish and International literature on implementation capacity and health promotion in schools in general [37–39].

### Variables

The participants’ attitudes towards SFSH were measured with the following item: “In your opinion, where do you think the school ought to be in two years in relation to SFSH?” The item had four response categories: 1) the school has implemented SFSH, 2) the school is in the process of implementing SFSH, 3) the school is not about to implement SFSH, and 4) do not know. This was to answer objectives one and two, which were recoded to reflect 1) favourable, 2) somewhat favourable, 3) not favourable or 4) indecisive attitudes towards SFSH. Covariates in the adjusted analysis were age, sex, educational attainment (short/VET, medium or long education) and years of employment at a VET school.

In the analysis examining the differences between the health promotion environment based on the managements’ attitude towards SFSH, only the principal manager’s answer to the same outcome variable was used. The item was dichotomized, and the schools were categorized as a school in favour of SFSH (response categories 1 and 2) or a school less in favour of SFSH (response categories 3 and 4). The principal manager’s attitude was used because it is a management decision as to whether a Danish VET school will implement SFSH. In some schools (*n* = 6), the responses from department managers were used as the principal management either did not answer the item (*n* = 4) or because there was disagreement between the principal managers (*n* = 2).

To explore the differences in Danish VET schools, the variables, type of school (agricultural, commercial, technical, or social healthcare), geographical location (by regions), and school size (< 800 students, 800–2000 students, or > 2000 students) were used in the analysis.

To explore the schools’ health promotion environments, this paper draws on variables from three themes: 1) policy and practice, 2) school facilities and 3) implementation capacity. The schools’ facilities were measured with ten items such as “There are facilities for indoor physical activity” (yes/no). Policy and practice were measured using 48 items such as “The school offers free breakfast at least once a week” (yes/no). Implementation capacity in relation to health promotion was measured using 20 items such as “The work with health promotion is based on a systematic use of knowledge” rated on a Likert scale from 1 to 5 (disagree-agree).

### Statistical analysis

The data were analysed using SPSS version 22. Principal managers and department managers were combined into one group and defined as “managers” to increase the power of the analysis. To assess the distribution of attitudes towards SFSH between managers and teaching staff, chi-square tests were used. To examine the variables that influence attitudes, multinomial logistic regression (MLR) was performed. The reference category for the outcome variable was a not favourable attitude. Unadjusted odds ratios (OR crude) and adjusted odds ratios (OR adjusted) with a 95% confidence interval (CI) from the MLR is reported.

Exploratory data analysis (EDA) was used to explore the differences in health promotion environments among the Danish VET schools according to management attitudes towards SFSH. The data were aggregated at the school level. Chi-square tests were used for categorical data and independent sampled t-tests for continuous items. Due to the questionnaire set-up, in some cases, the analysis was based on answers from either the management, the teaching staff, or both. Explanatory variables with less than 40 answers were excluded (11 items), and in the analyses on implementation capacity, the respondents who had answered “do not know” were excluded.

It should be noted that *p* < .1 (two-tailed) was considered a trend, and *p* < .05 was considered statistically significant. A sole focus on a traditional level such as .05 can fail to detect important variables [40]; therefore, trends are also reported due to the exploratory nature of this study.

## Results

### Participants

Data from 72 out of 87 (82%) Danish VET schools were obtained. Overall, a total of 570 participants answered the questionnaire (75 principal managers, 117 departmental managers and 278 teaching staff members). Participants characteristics are shown in Table 1. The number of respondents per school varied from 1 to 45 respondents. The participants who did not answer the item about attitude towards implementing SFSH were excluded (*n* = 125); thus, 445 participants (150 managers and 295 teaching staffs) distributed across 71 Danish VET schools (range 1–45) were included in this study. A non-response analysis showed no differences between the participating and non-participating schools based on geographical location, type of school and school size (*p* > .05).

**Table 1:** Participant characteristics (managers *n* = 150, teaching staff *n* = 295)

	Managers		Teaching staff			Total	
	n (%)	mean (SD)	n (%)	mean (SD)	*p*	n (%)	mean (SD)
Total	150 (33.7%)	**–**	295 (66.3%)	**–**		445 (100%)	**–**
Female	68 (45.3%)	**–**	159 (53.9%)	**–**	0.054	227 (51.0%)	**–**
Medium or long education	99 (66.0%)	**–**	59 (20.0%)	**-**	<0.001	158 (35.5%)	**–**
Age (in years)	**–**	50.43 (7.3)	**–**	45.85 (8.9)	<0.001	–	47.39 (8.7)
Years employed in a VET school	**–**	14.95 (8.9)	**–**	9.05 (8.0)	<0.001	–	11.04 (8.8)

*Notes:* The test-values are based on chi-square tests and independent sampled t-test

*VET* Vocational education and training, *SD* Standard deviation

### Attitudes towards SFSH

In both the crude (chi^2^ = 13.61, *p* = .003, Table 2) and adjusted analysis (Table 3), it was found that teaching staff had a more favourable attitude than managers towards SFSH. The adjusted analyses showed that the teaching staff were more than two times more likely than managers to have a favourable attitude towards SFSH (OR: 2.08, CI: 1.13–3.82, *p* = .018) compared with a not favourable attitude (Table 3).

**Table 2:** Attitude towards SFSH in Danish vocational schools, managers (*n* = 150) and teaching staff (*n* = 295)

	Managers	Teaching staff		Total
	n (%)	n (%)	*p*	n (%)
Attitude towards SFSH			0.003	
Favourable attitude	33 (22%)	98 (33.2%)		131 (29.4%)
Somewhat favourable attitude	31 (20.7%)	34 (11.5%)		65 (14.6%)
Less favourable attitude	64 (42.7%)	102 (34.6%)		166 (37.3%)
Indecisive attitude	22 (14.7%)	61 (20.7%)		83 (18.7%)

*Notes:* The test-values are based on chi-square test

*SFSH* Smoke-free-school-hours

**Table 3:** Variables which influence attitudes towards SFSH, crude and adjusted (*n* = 445)

	Favourable attitude				Somewhat favourable attitude				Indecisive attitude			
	OR crude (95% CI)	*p*	OR adjusted (95% CI)	*p*	OR crude (95% CI)	*p*	OR adjusted (95% CI)	*p*	OR crude (95% CI)	*p*	OR adjusted (95% CI)	*p*
Categorical variables												
*Employed as*												
Management	*REF*		*REF*		*REF*		*REF*		*REF*		*REF*	
Teaching staff	1.86 (1.13–3.08)	0.015	2.08 (1.13–3.82)	0.018	0.69 (0.39–1.23)	0.205	0.64 (0.31–1.33)	0.229	1.14 (0.98–3.10)	0.061	1.38 (0.69–2.78)	0.360
*Sex*												
Male	*REF*		*REF*		*REF*		*REF*		*REF*		*REF*	
Female	2.33 (1.46–3.72)	*p* < 0.001	2.36 (1.45–3.83)	0.001	3.43 (1.87–6.29)	*p* < 0.001	3.53 (1.89–6.61)	*p* < 0.001	1.76 (1.03–2.99)	0.038	1.58 (0.91–2.74)	0.101
*Educational attainment*												
Short or vocational education	*REF*		*REF*		*REF*		*REF*		*REF*		*REF*	
Medium or long education	0.97 (0.60–1.58)	0.916	1.30 (0.73–2.24)	0.385	1.50 (0.84–2.69)	0.174	1.14 (0.57–2.29)	0.703	0.85 (0.48–1.15)	0.570	0.99 (0.52–1.90)	0.991
Continuous variables												
Age	1.01 (0.99–1.04)	0.348	1.05 (1.01–1.09)	0.011	1.02 (0.99–1.06)	0.224	1.06 (1.01–1.11)	0.010	0.99 (0.96–1.02)	0.327	1.03 (0.99–1.07)	0.205
Years of employment in a VET school	0.99 (0.96–1.01)	0.255	0.98 (0.94–1.01)	0.202	0.98 (0.95–1.01)	0.243	0.95 (0.91–0.99)	0.025	0.96 (0.92–0.99)	0.007	0.95 (0.91–0.99)	0.019

Notes: REF: Reference category. The reference category is: Not favourable attitude (not shown)

*SFSH* Smoke-free-school-hours, *MLR* Multinomial logistic regression, *CI* Confidence interval, *OR* Odds ratio

### Variables that influence attitudes towards SFSH

Being female (OR: 2.36, CI: 1.45–3.83, *p* = .001) and increasing age (OR: 1.05, CI: 1.01–1.09, *p* = .007) further increased the odds of having a favourable attitude towards SFSH (Table 3). Similar results on sex and age were found in relation to a somewhat favourable attitude. Decreasing years of being employed at a VET school decreased the odds of having a somewhat favourable attitude (OR: 0.95, CI: 0.91–0.99, *p* = .025) and an indecisive attitude (OR: 0.95, CI 0.91–0.99, *p* = .019) compared to a not favourable attitude.

### Differences in health promotion environments between schools in favour and less in favour of SFSH

School characteristics from the 72 included VET schools are shown in Table 4. A trend towards schools in favour of SFSH having more health promotion facilities, policy and practice, was identified. Overall, 60 out of 67 items (89.6%) were in the hypothesized direction, with nine items showing a significant association (*p* < .05), two items showing a trend for a positive association (*p* < .1) and only one item showing a trend for a negative association (p < .1). For significant associations, see Table 5.

**Table 4:** Characteristics of schools in favour and schools less in favour towards SFSH

	Schools in favour of SFSH	Schools less in favour of SFSH	Total
	n (%)	n (%)	n (%)
Total	29 (40.8%)	42 (59.2%)	71 (100%)
Geographical location			
Capital Region of Denmark and Region Zealand	11 (51,8%)	10 (48,2%)	21 (14,8%)
Region of Southern Denmark	10 (45.5%)	12 (54.5%)	22 (31%)
Central and North Denmark Region	8 (24,3%)	20 (75,8%)	28 (19,3%)
Type of school			
Agriculture, food and Experiences	3 (42.8%)	4 (57.2%)	7 (9.9%)
Social and healthcare service	12 (75.0%)	4 (25.0%)	16 (22.5%)
Office, commerce and business	5 (22.7%)	17 (77.3%)	22 (31%)
Technology, construction and Transport	9 (34.6%)	17 (65.4%)	26 (36.6%)
School size			
<800 students	4 (19.0%)	17 (81.0%)	21 (29.6%)
800–2000 students	13 (52.0%)	12 (48.0%)	25 (35.2%)
>2000 students	7 (41.2%)	10 (58.8%)	17 (23.9%)
*Missing*	5 (62.5%)	3 (37.5%)	8 (11.3%)

*SFSH* Smoke-free-school-hours

**Table 5:** Facilities, policies and activities, and implementation capacity by schools in favour and less in favour of SFSH^a^

	Schools in favour of SFSH			Schools less in favour of SFSH			
	n	%	mean (SD)	n	%	mean (SD)	*p*
Total	29	40.8%		42	59.2%		
Facilities							
Vending machines, sale of soda or other sugary drinks	24	39.0%	**–**	26	59.0%	**–**	0.030
Vending machines, sale of sugary or fatty foods	24	24.0%	**–**	26	41.0%	**–**	0.037
Changing room facilities	24	71.0%	**–**	26	44.0%	**–**	0.004
Shower facilities	24	66.0%	**–**	26	43.0%	**–**	0.016
Student canteen	20	92.5%	**–**	22	100%	**–**	0.083
Policy and practice							
Offering free breakfast at least once a week	29	29.0%	**–**	42	10%	**–**	0.025
Offering individual smoking cessation counselling for students	20	17.0%	**–**	22	4.0%	**–**	0.047
Physical activity is integrated in the practical part (basic programme)	29	64.0%	**–**	42	41%	**–**	0.029
Physical activity is integrated in the practical part (main programme)	29	58.0%	**–**	42	33.0%	**–**	0.015
Implementation capacity							
Health promotion work is based on systematic use of knowledge	27	**–**	3.4^a^ (0.9)	39	**–**	2.9^a^ (0.9)	0.018
Management has created shared goals and direction for the school’s work with health promotion	28	**–**	3.1^a^ (0.9)	41	**–**	2.7^a^ (0.9)	0.085
Time is a barrier do to health promotion	28	**–**	2.6^a^ (0.7)	41	**–**	2.9^a^ (0.8)	0.053

*Notes:* ^a^ Only significant and trends are presented. ^a^ Items were measured on a Likert scale, ranged from 1 to 5

*SFSH* Smoke-free-school-hours, *SD* Standard deviation

## Discussion

This study is, to our knowledge, the first to investigate attitudes among managers and teaching staff towards implementing SFSH in a VET school setting. Attitudes towards SHSH among managers and teaching staff are important to address in the pursuit of implementing more restrictive tobacco policies in VET schools.

In this study, we found that the teaching staff in Danish VET schools had a more favourable attitude towards SFSH compared to the management, which is opposite to our hypothesis and that found in the scholarly literature on the health promoting school approach. The health promoting school approach involves a complex dynamic of group behaviours and system changes within a school by employees, students and external stakeholders, and it has been argued that the primary function of management is to stimulate readiness and motivation for change, to act as a role model and to incorporate practices and structures that can facilitate organizational development and change [41]. Thus, implementing new health promotion practices, e.g., SFSH, in organizations is mostly a process facilitated by management and not the other way around (bottom-up decision-making), even though teaching staff and student participation is important for successful implementation [41, 42]. Due to a general higher educational attainment among managers than teaching staff (medium- or long education, managers: 66%, teaching staff: 20%), a more positive attitude towards SFSH was expected as it has been found that high socioeconomic status (SES) groups in general are more positive and prone to support health promotion initiatives than people from lower SES groups [43, 44].

A possible explanation for the finding in our study is that aspects such as SFSH, and more broadly restrictive tobacco policies directed towards children and youth in Danish society, has recently gained support and attention from civil society, while the decision makers and politicians lack the will to enforce regulation [45]. Recently, the second largest supermarket chains have agreed to hide cigarettes behind the counter [46], and the Smokefree Future partnership aims to reach a maximum of 5% of adult smokers and ensure no children and adolescent smokers by 2030 in Denmark [45]. Such voluntary initiatives may have a great impact on public opinion [45], perhaps even more in lower SES groups, into which teaching staff at VET schools can be categorized. A reason for the more negative attitude among managers may be based on the presumption that both teaching staff and students, who both present very high smoking prevalence, will oppose the implementation of SFSH and as the schools are under pressure due to reforms and financial cuts [31], they judge it as unlikely that the schools will have implemented SFSH within 2 years.

Our study suggests that teaching staff is either more positive or at least more ready to implement restrictive tobacco policies than management. Despite the fact that teaching staff have a positive attitude towards SFSH, it is important to bear in mind that they may not be ready to support the actual implementation of SFSH. A research-based report based on data from the same study showed that teaching staff in general believe they have little competence and motivation to work with tobacco prevention [29].

The results of this study also suggest that certain demographic characteristics increased the odds for having a more favourable attitude towards implementing SFSH, especially sex and age. Females were more likely to have a favourable attitude towards implementing SFSH compared to men. This is consistent with the previous research suggesting that women are more likely to engage in health-related issues compared to men [47]. Most managers and teaching staff in “social and healthcare service” schools are female – and 75% of these schools were in favour of implementing SFSH. As it was not possible to adjust for type of school in the analysis due to a lack of power, it is likely that the results on sex are associated with school type. Social and healthcare service have a clear focus on health as part of their educational programme, and it seems logical that these schools and their managers would have a more favourable attitude towards SFSH. Qualitative findings support this by augmenting that schools view it at their responsibility to prepare students for future jobs where smoke-free environments are enforced, e.g., hospitals or kindergartens [17]. This study was the first to show that increasing age is associated with a more favourable attitude towards SFSH; however, especially given the exploratory approach adopted in this study, this finding must be replicated in future studies.

VET schools in favour of SFSH seem to have more health promotion policies, practices, facilities and implementation capacity in general, as almost all tests (89.6%) pointed in the same direction. However, only a few analyses led to significant differences or trends. Based on our study, it cannot be concluded that there is a causal association between managements’ attitudes towards SFSH and health promotion initiatives. Though, our analysis suggests an association between being a principal manager who prioritizes working with health promotion in general and prioritizing SFSH. Working with health promotion initiatives in general might build the capacity [42] or organizational readiness [48] to implement strategies such as SFSH. As such, schools that already have a clear health promotion profile and practice are ‘more ready’ than schools that have not yet, or have not for long, been working with health promotion activities. Thus, in some schools, SFSH can be more easily integrated and sustained in practice than in others. Some schools might need support in becoming aware of their role and responsibility as health promotion agents, and school management must prioritize this by establishing health promotion initiatives. In this case, it might be an appropriate strategy to identify schools with a long history of working with health promotion and encourage them to implement SFSH as they may be more ready. When a critical mass of schools has implemented SFSH, the rest (or the politicians) will be more likely to follow [49].

### Limitations

The findings from this cross-sectional study should be interpreted while keeping its limitations in mind.

The outcome item was formulated as follows: “In your opinion, where do you think the school ought to be in two years in relation to SFSH?”. A weakness in this study was that the management might be contemplating and balancing the implementation of SFSH in relation to the emergent school context, available time and economic resources, and on this basis, conclude that implementation within a two-year time frame is not realistic. SFSH is still a new concept in Denmark, and, therefore, it is a difficult idea to comprehend among people who are not familiar with the tobacco policy. Some participants might have confused SFSH with the less comprehensive “smoke-free school grounds”, which is a more common tobacco policy in Danish educational institutions. Although the questionnaire included a short description of SFSH entailment, it is a new concept that might have been misunderstood by some.

A total of 125 respondents did not answer the outcome variable and, towards the beginning of the questionnaire, they stated that the school had already established SFSH; these respondents were therefore excluded. A sensitivity analysis showed that the excluded respondents had worked at a VET school for a longer time compared to the included respondents (*p* = .014); otherwise, no differences in the excluded respondents compared to the included respondents were detected. Another possible explanation for the difference between managers’ and teaching staff’s attitudes is that department managers deliberately selected teaching staff with more favourable attitudes towards health promotion, hence creating a selection bias. In conclusion, there are various possibilities that could explain the unexpected difference in teaching staff’s and management’s attitudes towards implementing SFSH. However, these limitations are unlikely to have altered the validity of the results significantly.

On the management level, the representativeness is high with the principal manager represented at all included schools. The teaching staff level was represented at 70% of the included schools, varying from 1 to 37 teaching staff individuals per school. This issue was addressed by conducting sensitivity analyses adjusting for the number of respondents per school, and it did not change the magnitude of the results. To retain power, we have not adjusted for this issue in the final model.

Attitudes and practical actions for implementing SFSH are an unexplored research field, which is why EDA was used to summarize the main characteristics in Danish VET schools. Despite that, this study was based on a representative sample of Danish VET schools (83%), adjusted analysis was not possible due to the small number of VET schools in Denmark (*n* = 87).

## Conclusion and implications

This study investigated attitudes towards implementing SFSH in a representative sample of Danish VET schools and found that the teaching teaching staff were more likely to have a favourable attitude towards SFSH compared to school managers. As managers must be conveyors in the process of implementing health promotion initiatives at schools, this study indicates the importance of reaching both managers and teaching staff in the act of implementing SFSH. This study further indicates that existing health promotion initiatives, including policies and practice, facilities, and implementation capacity were associated with a favourable attitude towards SFSH among the principal managers. Decisions about health promotion initiatives are more likely to be made and successfully implemented if they align with school values and practices, thus implementing other health promotion activities and policies might be an important first step to establish readiness to implement SFSH. This study further highlights the need to include both the management and teaching staff in the development and implementation of health promotion initiatives to better align their attitudes.

## Data Availability

The datasets used and/or analysed during the current study are available from the corresponding author on reasonable request.

## Abbreviations

EDA: Exploratory data analysis
FCTC: Framework Convention on Tobacco Control
MLR: Multinomial logistic regression
SFSH: Smoke-free-school-hours
VET: Vocational education and training
WHO: World Health Organization

## References

[1] Stewart ST, Cutler DM, Rosen AB (2009). Forecasting the effects of obesity and smoking on U.S. life expectancy. N Engl J Med.

[2] Drope J, Schluger N, Cahn Z (2018). The tobacco atlas.

[3] Yach D, Wipfli H (2006). A century of smoke. Annals of Tropical Medicine & Parasitology.

[4] de Looze M, ter Bogt T, Hublet A (2013). Trends in educational differences in adolescent daily smoking across Europe, 2002-10. The European Journal of Public Health.

[5] Haug S, Schaub MP, Salis Gross C, et al. Predictors of hazardous drinking, tobacco smoking and physical inactivity in vocational school students. BMC Public Health. 13. Epub ahead of print December 2013. 10.1186/1471-2458-13-475. 10.1186/1471-2458-13-475 PMC3658930 23672294

[6] Bendtsen P, Mikkelsen SS, Tolstrup JS. Ungdomsprofilen 2014: sundhedsadfærd, helbred og trivsel blandt elever på ungdomsuddannelser. Statens Institut for Folkesundhed, 2015.

[7] Foley P. The socio-economic status of vocational education and training students in Australia. Adelaide, S. Aust.: National Centre for Vocational Education Research; 2007.

[8] The Danish Ministry of Education. Tal der taler 2009: uddannelsesnøgletal 2009. Kbh., 2010.

[9] Panatto D, Amicizia D, Domnich A (2013). Tobacco smoking among students in an urban area in northern Italy. J Prev Med Hyg.

[10] VanKim NA, Laska MN, Ehlinger E, et al. Understanding young adult physical activity, alcohol and tobacco use in community colleges and 4-year post-secondary institutions: a cross-sectional analysis of epidemiological surveillance data. BMC Public Health. 10. Epub ahead of print December 2010. DOI: 10.1186/1471-2458-10-208. 10.1186/1471-2458-10-208 PMC2876104 20420678

[11] Chatterjee N, Kadam R, Patil D, et al. Adherence to the tobacco-free school policy in rural India. Asian Pac J Cancer Prev Epub ahead of print September 2017. DOI: 10.22034/APJCP.2017.18.9.2367. 10.22034/APJCP.2017.18.9.2367 PMC5720638 28950680

[12] Lipperman-Kreda S, Paschall MJ, Grube JW (2009). Perceived enforcement of school tobacco policy and adolescents’ cigarette smoking. Prev Med.

[13] Main C, Thomas S, Ogilvie D, et al. Population tobacco control interventions and their effects on social inequalities in smoking: placing an equity lens on existing systematic reviews. BMC Public Health; 8. Epub ahead of print December 2008. DOI: 10.1186/1471-2458-8-178. 10.1186/1471-2458-8-178 PMC2412872 18505545

[14] Plaspohl SS, Parrillo AV, Vogel R (2012). An assessment of America’s tobacco-free colleges and universities. J Am Coll Heal.

[15] Christensen Tabita Maria, Møller Lisbeth, Jørgensen Torben, Pisinger Charlotta (2012). The impact of the Danish smoking ban on hospital admissions for acute myocardial infarction. European Journal of Preventive Cardiology.

[16] Jarlstrup NS, Juel K, Pisinger CH (2018). International approaches to tobacco use cessation programs and policy in adolescents and young adults: Denmark. Current Addiction Reports.

[17] Rasmussen KH, Hansen AV, Klinker CD (2018). Udbredelse af røgfri skoletid på erhvervsskoler - En forundersøgelse til en effektiv tobaksforebyggelsesindsats på erhvervsskoler.

[18] retsinformation.dk. Lov om røgfri miljøer [Internet]. 2007 [cited 2018 Sep 12]. Available from: https://www.retsinformation.dk/forms/r0710.aspx?id=11388#K2.

[19] Agaku IT, Obadan EM, Odukoya OO (2015). Tobacco-free schools as a core component of youth tobacco prevention programs: a secondary analysis of data from 43 countries. The European Journal of Public Health.

[20] Vestbo J, Pisinger C, Bast LS, et al. Forebyggelse af børn og unges rygning. Hvad virker? København: Vidensråd for Forebyggelse, 2018.

[21] Andersen S, Rod MH, Ersbøll AK (2016). Effects of a settings-based intervention to promote student wellbeing and reduce smoking in vocational schools: a non-randomized controlled study. Soc Sci Med.

[22] Emami H, Soha Rezai Shiraz A, Naseri G (2011). The determinants of high school students smoking habits with special focus on teachers smoking in Iran: a population based study.

[23] Trinidad DR, Gilpin EA, Pierce JP (2005). Compliance and support for smoke-free school policies. Health Educ Res.

[24] *Aftale om Bedre og mere attraktive erhvervsuddannelser*. Denmark: Regeringen (Socialdemokraterne og Radikale Venstre), Venstre, Dansk Folkeparti, Socialistisk Folkeparti, Konservative Folkeparti og Liberal Alliance, February 2014.

[25] Cutler DM, Lleras-Muney A (2010). Understanding differences in health behaviors by education. J Health Econ.

[26] The Danish Ministry of Education. Improving Vocational Education and Training – overview of reform of the Danish vocational education system [Internet]. Copenhagen; 2014. Available from: https://www.uvm.dk/publikationer/engelsksprogede/2014-improving-vocational-education-and-training.

[27] Andersen S, Riis N, Nygart V, et al. Rygning på erhvervsskoler - det skal være federe at være ikkeryger. 1. udgave, København: Vidensråd for Forebyggelse, 2018.

[28] Egan KK, Pisinger VSC, Christensen AI, et al. Rygevaner blandt gymnasie- og erhversskoleelever. Statens Institut for Folkesundhed, 2017.

[29] Hansen AV, Klinker CD (2017). Danske erhvervsskolers - sundhedsfremmende indsatser og implementeringskapacitet.

[30] Ingholt L, Sørensen BB, Andersen S, et al. How can we strengthen students’ social relations in order to reduce school dropout? An intervention development study within four Danish vocational schools. BMC Public Health; 15. Epub ahead of print December 2015. DOI: 10.1186/s12889-015-1831-1. 10.1186/s12889-015-1831-1 PMC4448564 25997429

[31] UVM. UVM. 2017 [cited 2018 Sep 12]. Available from: https://www.uddannelsesstatistik.dk/Pages/Topics/23.aspx.

[32] DEA. De voksnes ungdomsuddannelser. Copenhagen: DEA, March 2014.

[33] European Centre for the Development of vocational training (Cedefop) (ed). Vocational education and training in Denmark: short description. Luxembourg: Publications Office of the European Union, 2012.

[34] deg.dk. Find skoler [Internet]. 2018 [cited 2018 May 23]. Available from: https://deg.dk/index.php?id=1915.

[35] deg.dk. Erhvervsskolerne i tal [Internet]. 2015. Available from: https://deg.dk/uddannelserne/erhvervsskolen-i-tal/.

[36] Israel M, Hay I. Research ethics for social scientists: between ethical conduct and regulatory compliance. London; thousand oaks, calif: Sage, 2006.

[37] Chilenski SM, Olson JR, Schulte JA (2015). A multi-level examination of how the organizational context relates to readiness to implement prevention and evidence-based programming in community settings. Evaluation and Program Planning.

[38] Flarup LH, Greve J, Søndergaard PH, et al. Grundforløb på erhvervsuddannelserne inden reformen: baselinemåling. KORA, 2015.

[39] Scholtens S, Middelbeek L, Rutz SI, et al. Differences in school environment, school policy and actions regarding overweight prevention between Dutch schools. A nationwide survey. BMC Public Health. 10. Epub ahead of print December 2010. 10.1186/1471-2458-10-42. 10.1186/1471-2458-10-42 PMC2823704 20109197

[40] Bendel RB, Afifi AA (1977). Comparison of stopping rules in forward ‘stepwise’ regression. J Am Stat Assoc.

[41] Rowling L, Samdal O (2011). Filling the black box of implementation for health-promoting schools. Health Educ.

[42] Durlak JA, DuPre EP (2008). Implementation matters: a review of research on the influence of implementation on program outcomes and the factors affecting implementation. Am J Community Psychol.

[43] Marmot Michael, Bell Ruth (2016). Social inequalities in health: a proper concern of epidemiology. Annals of Epidemiology.

[44] Marmot M, Friel S, Bell R (2008). Closing the gap in a generation: health equity through action on the social determinants of health. Lancet.

[45] WHO. Capacity assessment on the implementation of effective tobacco control policies in Denmark. Denmark: WHO, January 2018.

[46] Coop Danmark [Internet]. [cited 2018 Dec 6]. Available from: https://coop-sundhed.s1.umbraco.io/.

[47] McIntyre E, Prior J, Connon ILC (2018). Sociodemographic predictors of residents worry about contaminated sites. Sci Total Environ.

[48] Weiner BJ. A theory of organizational readiness for change. *Implementation Science*; 4. Epub ahead of print December 2009. DOI: 10.1186/1748-5908-4-67. 10.1186/1748-5908-4-67 PMC2770024 19840381

[49] Dearing JW (2009). Applying diffusion of innovation theory to intervention development. Res Soc Work Pract.

